# Macro-Modelling of Laser Micro-Joints for Understanding Joint Strength in Electric Vehicle Battery Interconnects

**DOI:** 10.3390/ma14133552

**Published:** 2021-06-25

**Authors:** Abhishek Das, Richard Beaumont, Iain Masters, Paul Haney

**Affiliations:** 1 WMG, The University of Warwick, Coventry CV4 7AL, UK; richard.beaumont@warwick.ac.uk (R.B.); I.G.Masters@warwick.ac.uk (I.M.); 2 Jaguar Land Rover Limited, Coventry CV3 4LF, UK; phaney@jaguarlandrover.com

**Keywords:** laser micro-welding, FE macro-model, thin sheet, joint strength, tensile performance

## Abstract

Laser micro-welding is increasingly being used to produce electrically conductive joints within a battery module of an automotive battery pack. To understand the joint strength of these laser welds at an early design stage, micro-joints are required to be modelled. Additionally, structural modelling of the battery module along with the electrical interconnects is important for understanding the crash safety of electric vehicles. Fusion zone based micro-modelling of laser welding is not a suitable approach for structural modelling due to the computational inefficiency and the difficulty of integrating with the module model. Instead, a macro-model which computationally efficient and easy to integrate with the structural model can be useful to replicate the behaviour of the laser weld. A macro-modelling approach was adopted in this paper to model the mechanical behaviour of laser micro-weld. The simulations were based on 5 mm diameter circular laser weld and developed from the experimental data for both the lap shear and T-peel tests. This modelling approach was extended to obtain the joint strengths for 3 mm diameter circular seams, 5 mm and 10 mm linear seams. The predicted load–displacement curves showed a close agreement with the test data.

## 1. Introduction

Electric vehicles (EVs), hybrid/plug-in hybrid electric vehicles (HEVs/PHEVs) are increasingly being used to replace traditional fossil fuel-based automotive vehicles in order to minimise the generation of greenhouse gases [1,2,3]. This recent uptake of electric vehicles demands a significant level of research and development in the fields of battery modelling and simulation, electrochemistry, thermal management and safety [3,4,5]. Similarly, advancement in manufacturing methods is equally important to achieve the increasing global demand for automotive battery packs. In general, an automotive battery pack for use in electric vehicles consists of a large number of individual battery cells held in a structural frame and electrically connected together in modules. Several modules are combined within a battery pack to achieve the desired power and capacity requirements [6]. Manufacturing of Li-ion battery packs demands a significant number of electrically conductive joints, such as welds between the battery tabs and cell terminal. These battery interconnect joints are integral parts of an electric vehicle battery pack. For example, a cylindrical cell-based module may contain several hundreds of cells, which must be connected at both the positive and negative terminals. Therefore, there is a need for both an efficient and reliable joining method to produce these large number of joints.

The choice of joining technique largely depends on the ability to satisfy several requirements including (i) joining dissimilar materials; (ii) joining highly conductive and reflective materials; (iii) overcoming the challenge of multi-layered stack-ups; (iv) obtaining high joint strength and low joint resistance; and (v) producing the joint with low heat input or high thermal fatigue resistance [7]. Research is being pursued to identify suitable joining techniques for building battery packs. One key factor which often drives the selection joining techniques is the cell type (i.e., cylindrical, pouch or prismatic cells) used to build the battery pack [7,8]. For example, in the cylindrical cell-based battery module, cell terminals and external tabs/busbar can be connected by adopting both current and emerging joining processes including ultrasonic wedge bonding, soldering, resistance spot/projection welding, pulsed micro-TIG welding or laser welding. Of these, laser welding provides several benefits including single-sided access, non-contact joining, low thermal input, high process speed and ease of automation. Laser welding is also being widely investigated for its suitability for dissimilar materials joining and has the potential to replace ultrasonic wedge bonding as the preferred welding process for cell joining.

The interconnection joints are an integral part of the battery module together with the cells and ancillary components. Structural FE models are being developed to represent the battery cells [9], battery modules, and subsequently the pack behaviour at micro and/or macro levels [10,11]. Mechanical modelling of these modules is important for safety performance studies to understand the behaviour in, for example in the event of a crash or module. However, battery interconnects are not extensively considered within these FE module models. Therefore, to replicate the accurate behaviour of battery modules and packs, modelling of the battery interconnect joints and subsequent integration with module models are necessary. One of the key industrial concerns for large scale simulations, e.g., full vehicle crash or whole battery pack/module compression is to develop macro-models having computational efficiency whilst achieving good agreement with a physical solution. In contrast, the joining requirements within the battery module are mainly micro-welds where thin sheets are to be welded. For example, Li-ion cylindrical cell casings are typically made of electrical grade steel of 300 µm average sheet thickness and the cell terminals are connected with copper-based tabs of the same thickness. Laser micro-welding is an efficient joining method to produce these electrical interconnects between copper and steel [12]. Several attempts have been made to model laser welded joints to predict the mechanical and metallurgical behaviours since obtaining a large experimental database is costly and time consuming. However, in-depth knowledge of process behaviour is vital to predicting the mechanical and microstructural properties of the laser joints. Based on an understanding of the laser joining process, researchers have tried to simulate the joining process as laser heat source modelling [13]. This allows prediction of several key weld characteristics including fusion zone geometry, temperature gradient, stress distribution and thermo-mechanical distortion [14,15,16]. However, modelling of the actual joining process is not trivial, as it includes the integration key nonlinear behaviours including mechanical, microstructural and thermal phenomena. Researchers have developed a three-dimensional finite element model in order to determine the temperature field and molten pool shape during the welding process [17,18]. Furthermore, several studies have been reported to determine the material properties, the stress–strain state in the joint, tensile strength and fracture behaviour [19,20,21]. Dal and Fabbro [22] provided an overview of the laser welding simulation where they categorised the simulation models into two kinds which are the thermo-mechanical simulations and the multi-physical simulations as a complete simulation is too large to be computed with one model. The first category aims to model the mechanical stress and strain due to the welding whereas the thermal and velocity fields are computed in the second category. Zain-ul-abdein et al. [23] investigated the effect of metallurgical phase transformations upon the residual stresses and distortion induced by laser beam welding in a T-joint configuration using the finite element method. Such three-dimensional weld pool geometry-based laser joint models or heat source are difficult to incorporate/place within full battery module/pack models. In general, in-depth micro-modelling is computationally expensive and not suitable for integration with large simulation models, such as automotive body-in-white or electric vehicle battery pack crash simulation. In contrast, computationally inexpensive macro-models can provide an effective solution for performing FE analysis for automotive applications. For example, Kuppuswamy et al. [24] studied coarse finite element modelling for predicting the impact strength of laser welds for automotive applications. They developed a substitute model of the weld where a simplified FE model was composed of the upper and lower flanges (modelled with shell elements) and solid weld element between the flanges. A similar approach was adopted by Chauffray et al. [25] and Arif [26] where the weld was modelled as solid brick elements placed within the coarse shell elements of the body-in-white sheet materials [27]. Utilising this macro-modelling approach, the strength of laser welds on car seats and subsequent failure prediction were obtained. In battery assemblies, laser micro-joints are useful to obtain the required connection between the cell terminal and tab. Trattnig and Leitgeb [11] emphasised the importance of joint strength and failure of joints during battery module modelling for crash safety simulation. Modelling the mechanical deformation of joints is equally as important as that of the casing, conductors, or isolators but as yet has received little attention. Therefore, in this study macro-modelling approach has been adopted to model the battery interconnects to allow easy integration with a larger simulation model, for example, a structural model of a battery module.

The aforesaid research gap is addressed in this paper by fulfilling the following objectives: (i) macro-modelling of laser micro-joints to evaluate the joint strength; (ii) experimental laser micro-joining to produce dissimilar joints; and (iii) verify and validate the model by performing lap shear and T-peel tests.

## 2. Materials and Experimental Procedures

### 2.1. Materials and Joint Configuration

**Figure 1:**
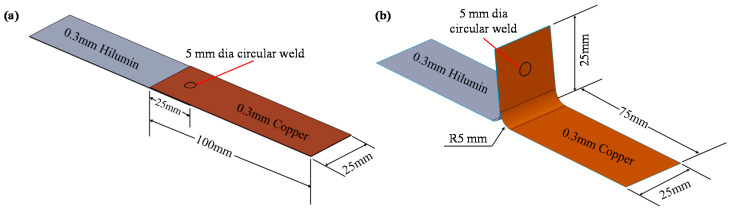
Schematic of micro-joint configurations having 5 mm diameter circular seam: (**a**) lap shear joint, and (**b**) T-peel joint.

In general, the Lithium-ion (Li-ion) cylindrical cell case is made of nickel-plated steel, such as Hilumin which is an electro nickel-plated, diffusion annealed steel strip for battery applications where low contact resistance and high corrosion resistance are needed. Nickel-plated copper strips are commonly used as tabs to connect the cylindrical cells to the busbar. Therefore, Hilumin and copper of 0.3 mm thickness were used in this experimental investigation and their corresponding chemical compositions are given in Table 1. To create the representative tab to cylindrical cell terminal interconnects, laser micro-welding was performed with the copper was placed on top of the Hilumin steel. The lap shear and T-peel samples were prepared with a 5 mm diameter circular seam weld was placed at the centre of the overlap region. Schematics of both lap shear and T-peel samples are shown in Figure 1.

**Figure 2:**
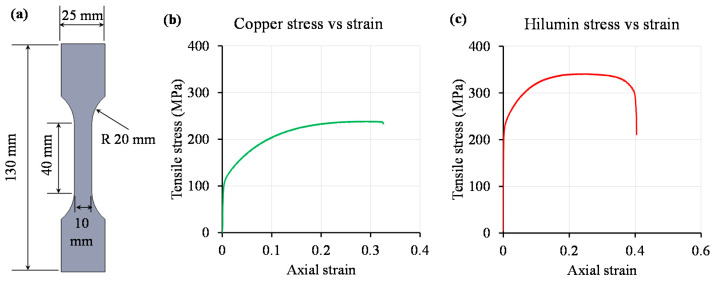
(**a**) Schematic of the tensile specimen, (**b**) stress–strain curve of the copper base material, and (**c**) stress–strain curve of the Hilumin steel base material.

The stress–strain behaviour of the parent materials was determined using a 40 mm gauge length sample tested at a quasi-static speed of 2 mm/min [28]. The geometry of the test pieces and the stress–strain curves of copper and Hilumin base materials of same 0.3 mm thickness are given in Figure 2. The tensile stress at yield (offset 0.2%) and maximum tensile stress of copper were 117 MPa and 239 MPa respectively whereas higher values were obtained for Hilumin steel (i.e., 217 MPa and 342 MPa). The physical and mechanical properties of copper and Hilumin used in this study are reported in Table 2.

### 2.2. Laser Welding System

**Figure 3:**
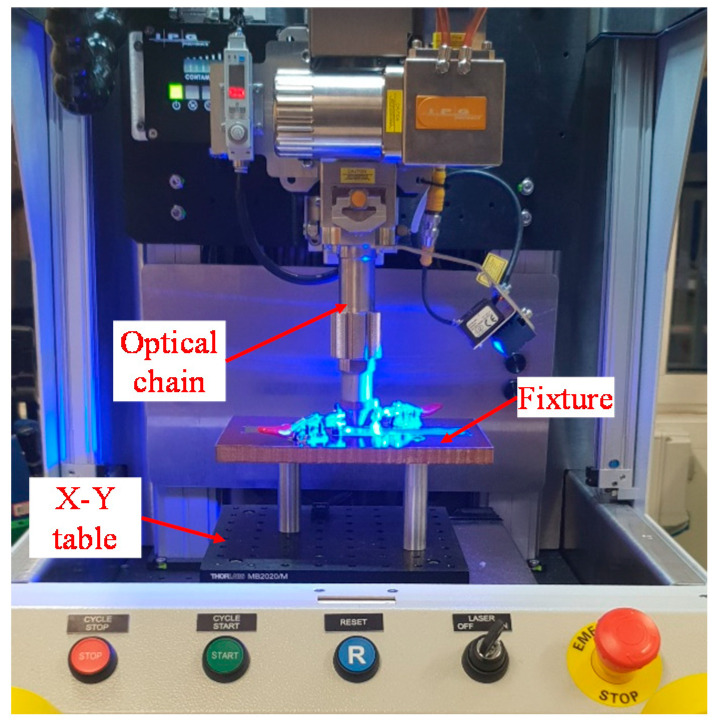
Laser welding setup with the fixture to ensure intimate contact.

Pulsed laser welding was carried out using an IPG 150W Quasi-CW IR laser (IPG Photonics, Oxford, MA, USA) having a maximum peak power of 1.5 kW. A wobble head was integrated within the optical chain to facilitate laser beam oscillation and the mode of operation was quasi-continuous wave (QCW) at an infrared (IR) wavelength of 1070 nm. The laser optics were mounted on an X-Y gantry system and the spot size at the focus was approximately 28 µm in diameter. A fixture was used to ensure intimate contact between the sheets by eliminating any part-to-part gap. The laser set-up with the fixture configuration is shown in Figure 3. In this experimental study, the laser beam was positioned perpendicular to the surface and focused on the top of the copper tab. The micro-welding was performed using argon shielding gas at a flow rate of 20 L/min. The wobble head was capable of producing wobble in various motions such as circular clockwise, circular counter-clockwise, infinity and figure of 8. For this investigation, a circular clockwise motion was selected. The laser welding process parameters with their corresponding ranges are given in Table 3. In the initial screening study, the parameter ranges were identified based on machine specification and welding requirements. The parameter variation was performed in two stages. Firstly, based on the joint strength obtained in the pilot experimentation, the wobble amplitude, wobble frequency and focus position were identified and then maintained at 0.4 mm, 400 Hz and on top surface respectively. Thereafter, process parameters such as laser power, pulse on time, pulse frequency and welding speed were chosen to produce the lap shear and T-peel test specimens to validate the macro-model approach of these laser micro-joints. Since welding speed is one of the important parameters to reduce the cycle time of the production process, the welding speed was kept at machine defined maximum level of 1500 mm/min. Using additional experiments and their results, the laser welding process parameters used in this study to produce good-weld are listed in Table 4.

### 2.3. Details of Specimen Preparation and Test Conditions

After carrying out the laser micro-welding, samples were tested to obtain the lap shear and T-peel strengths. The lap shear and T-peel tests were performed using an Instron 3367 static test frame (Instron, Wycombe, England) with a 30 kN load capacity and cross-head speed of 2 mm/min and 20 mm/min, respectively. A higher test speed was applied to perform the T-peel tests in order to minimise the unexpected dynamic effect for weld failure [29]. The peak loads obtained from the lap shear and T-peel tests, as well as load–grip displacement profiles, were recorded to evaluate the mechanical strength of the weld and further used for macro-model validation. For each test condition, three samples were prepared for measurement of the tensile strengths to obtain consistent and reliable results.

## 3. FE Macro-Modelling Approach

**Figure 4:**
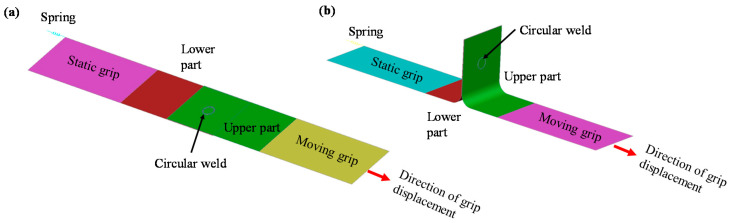
Schematic of test model set-ups: (**a**) lap shear simulation model and (**b**) T-peel simulation model.

The parent materials were modelled as sheet metal parts using shell elements. Solid brick elements provide more accurate results in comparison with shell elements as they are more suitable to calculate stress in all the three directions. Therefore, a solid hexahedral mesh was used to model the weld zone. The LS-DYNA platform [30] was used to develop the FE model for representing the mechanical behaviour and failure modes of the laser-welded micro-joint. The lap shear and T-peel test specimen model representations are given in Figure 4 showing where the circular seam was placed.

**Figure 5:**
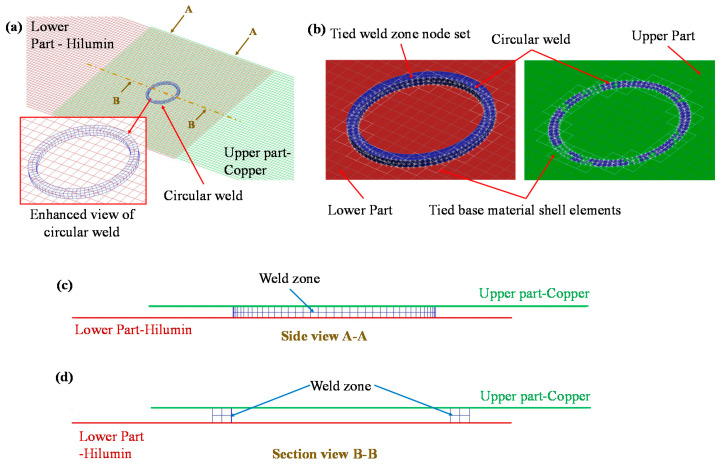
Details of the mesh model: (**a**) a closer look at the joint, (**b**) illustration of contact modelling, (**c**) side view of the weld A-A, and (**d**) section view at the centre of the weld B-B.

It can be observed that an elastic spring was added to the static grip of both the lap shear and T-peel specimen to replicate the load cell behaviour during the experimental tests. Additionally, the model was composed of static grip, moving grip, lower part, upper part and circular weld. In line with experimental measurement, the static grip and lower deformable parts were modelled with Hilumin material properties whereas the upper deformable part and moving grip were assigned the copper material properties. Standard Belytschko-Tsay membrane formulation was used to model shell elements used in the four sheet metal parts (i.e., static grip, moving grip, lower part, and upper part). An initial mesh sensitivity analysis was performed to select the appropriate mesh size and it was observed that a rectangular mesh size of 0.5 mm was suitable for both the lap shear and T-peel modelling since a further reduction in element size did not have a significant impact on the results. As mentioned earlier, the weld was modelled as a solid hexahedral mesh, however, a single layer of solid elements was not sufficient to obtain a stable model as excessive deformation with early element deletion was obtained. To overcome this issue, a 2 × 2 layer of solid elements varying in size from 0.15 mm to 0.2 mm was sufficient to replicate the welding behaviour (i.e., load–displacement behaviour and a further increase in the number of layers did not have any impact on the load–displacement curve characteristics). A close-up of the joint mesh for the lap shear model is shown in Figure 5a. The model was composed of 13,573 nodes, 12,500 shell elements and 320 solid elements. To place the weld in between the upper and lower part, two contact-based constraints were defined for (i) the upper part and the weld zone top surface, and (ii) the weld zone bottom surface and lower part. These contacts were introduced by using LS-Dyna contact treatment card *TIED_SURFACE_TO_SURFACE. In this contact model, the weld zone was assigned to the slave side and the larger base material parts (i.e., upper and lower parts) were assigned to the master side to achieve uniformity in the model (i.e., smaller part was defined as a slave whereas the bigger part was assigned as master). The node-set of the weld was tied with the shell element set of the base material as shown in Figure 5b. Therefore, this macro-modelling approach is useful for easy integration of the weld zone with other structural models since the weld can be placed easily without modifying the geometry or mesh. This makes the macro-modelling approach useful and adaptable for electric vehicle battery crash simulation or other structural integrity simulation.

The static and moving grips were modelled as rigid bodies where the static grip was constrained with all 6 degree of freedoms (DOFs) and the moving grip was displaced in the loading direction with a velocity profile. To simulate the base material and weld zone, five different material data cards were used which are Hilumin rigid body for static grip, Hilumin deformable body for the lower part, copper rigid body for moving grip, copper deformable body for upper part and fusion zone material card for weld seam (see Figure 4). These material cards were introduced using LS-Dyna material card *MAT_020: RIGID for rigid-body modelling whereas *MAT_024: Piecewise Linear Plasticity material model was used to model the material properties of the base deformable material as well as the weld fusion zone. The basic common material properties are listed in Table 2. The stress–strain behaviour of the base material was incorporated into the material model using a tabulated approach available in LS-DYNA and isotropic hardening effect for all the materials was assumed. The base material properties were obtained from mechanical testing whereas the fusion zone characterisation was more complex since it was hard to obtain the fusion zone properties. However, information on the microhardness of the fusion zone can be obtained. Therefore, the hardening curve of the fusion zone was derived by combining the base materials and microhardness test values (refer to Section 4.2 for details). Additionally, the fracture strain was also important to obtain the fracture at the required grip displacement. This fracture strain was selected to initiate fracture in the simulations when the experimentally measured displacement-to-failure was reached.

## 4. Results and Discussion

### 4.1. Joint Fusion Zone Characteristics

**Figure 6:**
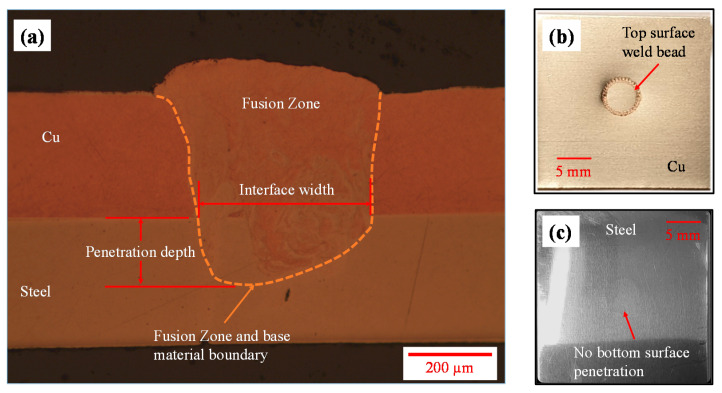
Laser micro-welded joint: (**a**) optical micrograph of the fusion zone, (**b**) top Cu surface weld bead, (**c**) bottom steel surface.

The laser micro-welding was performed using the process parameters described in Section 2.2 and Table 4. The optical micrograph of the weld fusion zone is shown in Figure 6a where the orange, dashed line represents the fusion zone and base material boundary. Two important fusion zone geometrical characteristics are penetration depth and interface width since these two criteria are often used to define the quality of the joint. Three repeats were performed to obtain the average penetration depth and interface width which were 105 µm and 392 µm respectively. As the laser micro-welding was conducted from copper to steel, the top surface of the weld bead and corresponding steel bottom surface are shown in Figure 6b,c respectively. Overall good surface appearance was observed and no detectable defect was noticed on the top surface of the weld stitch. Full penetration is not ideal for battery application as it can produce cracks leading to electrolyte leakage or potential thermal run away. This emphasises the importance of controlling penetration depth for low thermal input. It can be observed in Figure 6c that no bottom surface penetration was obtained in the steel surface. Therefore, the laser micro-welds obtained using the process parameters in this study were considered of good quality and further used for lap shear and T-peel tests. In addition, microhardness measurements were obtained as welding changed the mechanical properties of the material in the fusion zone. As discussed earlier, mapping the mechanical properties of the fusion zone is challenging whereas obtaining microhardness values is relatively straightforward. Microhardness analysis was conducted to determine the microhardness of parent materials and the change in microhardness within the fusion zone. The average microhardness of copper and steel as-received materials were 66.7 HV0.3 and 100.45 HV0.3 whereas the microhardness of the upper fusion zone was relatively higher as 76.7 HV0.3. This change in microhardness value was used to define the weld zone properties for simulation.

### 4.2. Macro-Modelling of Joint Strength and Failure Prediction

**Figure 7:**
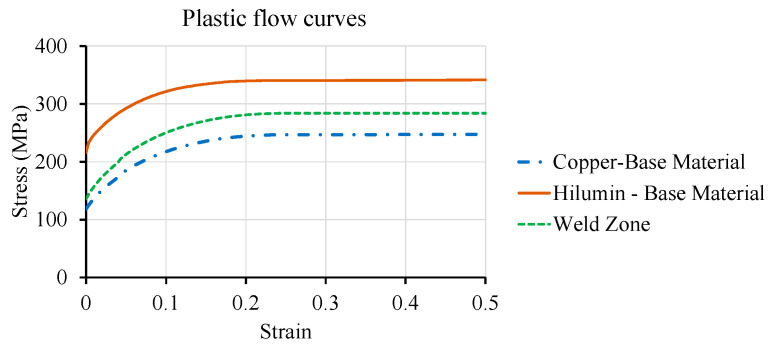
Plastic flow curves of base materials (i.e., copper, Hilumin steel) and weld zone.

Laser micro-welded joint strength and failure prediction are considered a high priority from a design perspective. Macro-modelling approach was adopted in this study without sacrificing the reliability of welding behaviour prediction. The base material properties were obtained from suppliers as well as by performing tensile tests. For example, the hardening curves of the copper and steel base material were obtained from the tensile tests. In contrast, fusion zone or weld zone hardening curves were derived from the base materials based on the hardness change. Therefore, the hardening curve for the weld zone was defined as $\sigma_{WZ}=\sigma_{BM}\times \left(HV_{WZ}/BM_{BM}\right)$. The copper plastic flow curve was chosen to represent the weld zone since the fusion zone was mostly composed of copper material and failure occurred within the copper part. As the average microhardness value of the upper weld zone was 1.15 times higher than the copper, the copper plastic flow curve was modified to obtain the weld zone curve. The hardening curves for the copper and steel base materials and weld zone is shown in Figure 7. The fracture strain was selected to initiate the fracture in the simulation at a grip displacement obtained from the experimental lap shear test. Additional material data and model specific details are listed in Table 5.

**Figure 8:**
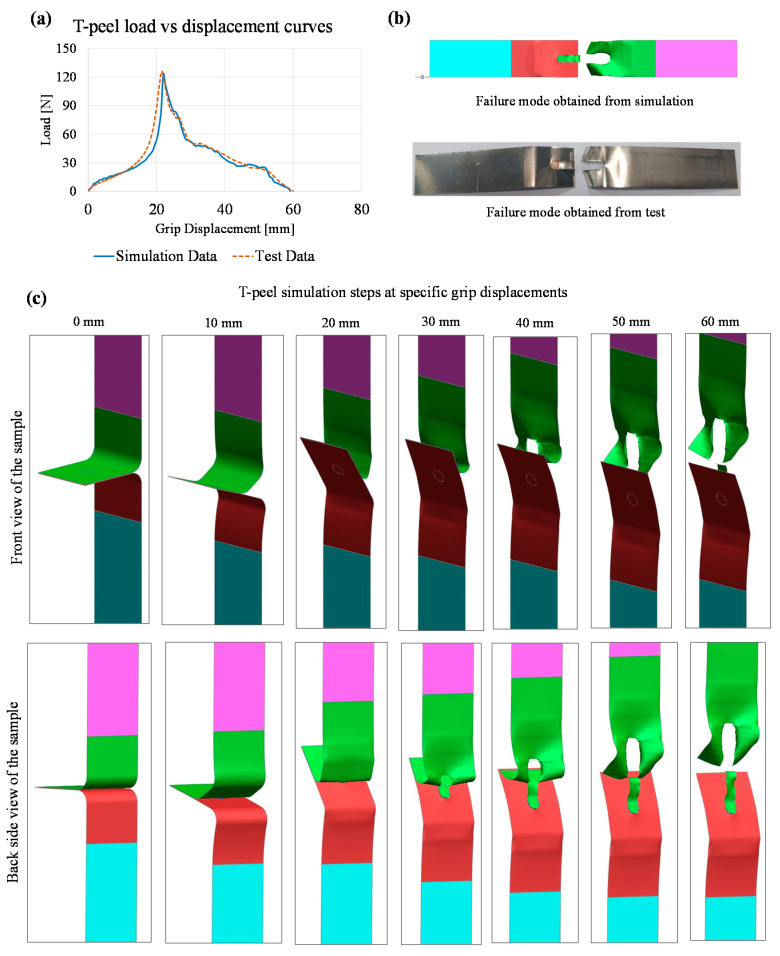
Simulation of T-peel test considering 5 mm diameter circular seam: (**a**) comparison of load–displacement curves obtained from simulation and tensile test, (**b**) failure modes obtained from simulation and test, and (**c**) sequence of images from the simulation at specific grip displacement.

**Figure 9:**
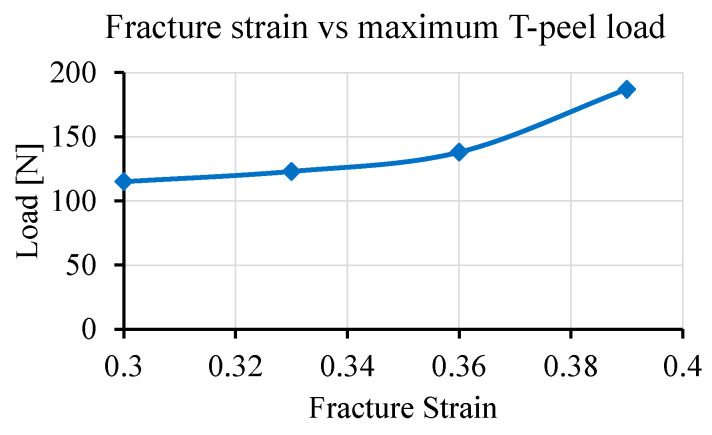
Impact of fracture strain on maximum T-peel load obtained by performing T-peel simulations.

The results obtained from the T-peel simulation are given in Figure 8. The maximum peak load obtained from the T-peel test was 123.27 N. Figure 8a shows that load–displacement curves obtained from the simulation and experimental T-peel tests were almost similar. The failure modes from simulation and experiment are shown in Figure 8b where the failure was initiated at the circumference of the weld and propagated by tearing of the base material. The weld nugget was strong enough to remain intact with the lower Hilumin sheet and failure was obtained in the copper sheet. The images from the deformation sequence at specific grip displacements are shown in Figure 8c. The fracture strain was an important criterion to obtain the maximum load from both the lap shear and T-peel tests. For example, a fracture strain-based sensitivity analysis was performed on T-peel strength analysis as it was observed that fracture strain had a substantial impact on obtaining the maximum T-peel load. Therefore, the fracture strain was varied for the upper copper material as the fracture was always obtained in the upper part. The sensitivity of this localised fracture strain used to initiate fracture at the desired load is plotted in Figure 9. It can be seen that the maximum T-peel load was increasing with the incremental increase in copper fracture strain. The localised fracture strain value of 0.33 gave the maximum T-peel load which was identical with the maximum T-peel load obtained from the experimental T-peel tests.

**Figure 10:**
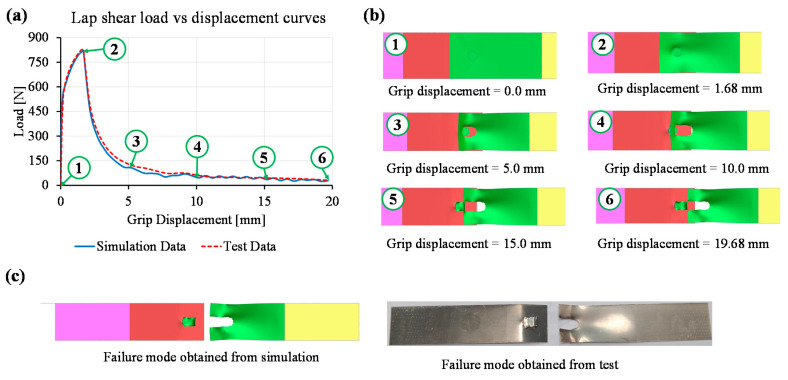
Simulation of lap shear test considering 5 mm diameter circular seam: (**a**) comparison of load–displacement curves obtained from simulation and tensile tests, (**b**) sequence of images from the simulation at specific grip displacement, and (**c**) failure modes obtained from simulation and test.

Similar to the T-peel test, the load–displacement curves obtained from the lap shear test and the corresponding simulation model are shown in Figure 10a. The maximum lap shear strengths obtained from the simulation and test were 820.4 N and 829.3 N. It is evident from the load–displacement curves that the macro-modelling of the laser micro-weld was in good agreement with the actual lap shear test. Selected images from the deformation sequence at specific grip displacements are shown in Figure 10b. It was observed that failure in the copper sheet was around the circumference of the weld and slowly propagated through the base material. Since the weld was attached with the lower sheet (i.e., steel) and failure was obtained in the upper sheet (i.e., copper) base material, the quality of weld was categorised as good-weld. Figure 10c confirms that the simulation model can predict the failure mode obtained from the lap shear test.

### 4.3. Validation of Maro-Model

**Figure 11:**
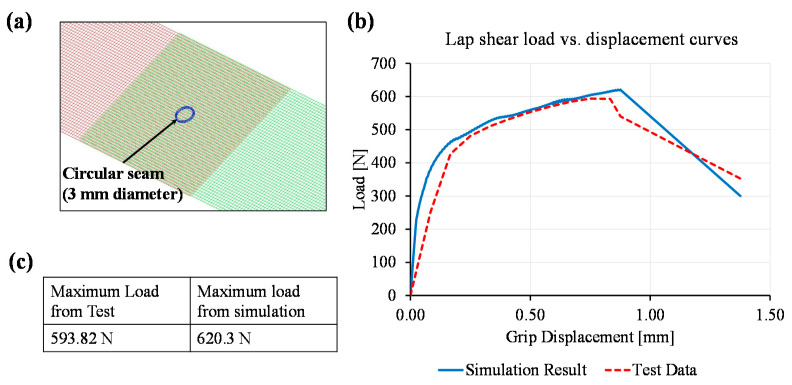
Simulation of lap shear test considering 3 mm diameter circular seam: (**a**) mesh representation of the seam, (**b**) enhanced view of load–displacement curved to show the load–displacement curves obtained from simulation and tensile tests, (**c**) maximum loads obtained from simulation and lap shear test.

The developed macro-model was validated using additional tests considering a 3 mm diameter circular laser micro-welded seam, as shown in Figure 11a. To obtain the maximum load and initiate fracture after the maximum load, the localised fracture strain was selected as 0.33 (i.e., based on the 5 mm diameter circular seam results). The load–displacement curves obtained from the lap shear test and corresponding simulation are plotted in Figure 11b and maximum load values are given in Figure 11c. The maximum load from the test was 593.82 N which was 26.98 N or 4.5% lower than the predicted load obtained from the simulation. Therefore, the macro-model gave a predictive load that was close to the actual test load. This validates the utilisation of the macro-model at an early stage of design.

## 5. Prediction of Linear Seam Joint Strength

**Figure 12:**
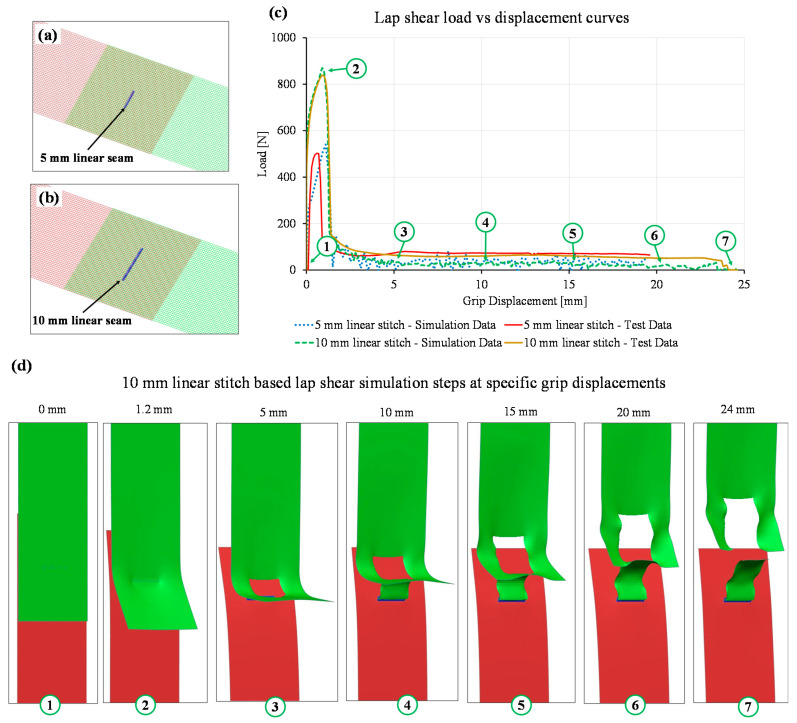
Simulation of linear stitch-based lap shear models: (**a**) mesh representation of 5 mm linear seam, (**b**) mesh representation of 10 mm linear seam (**c**) comparison of load–displacement curves obtained from simulation and tensile tests of 5 mm and 10 mm laser micro-welds, and (**d**) sequence of images from the simulation at specific grip displacement obtained from the simulation of the 10 mm linear seam.

The macro-model was used to predict the joint strength for linear stitches of 5 mm and 10 mm length as shown in Figure 12a,b. The same laser welding process parameters, as described in Section 2.2, were used to produce these linear welds. The linear stitches were modelled using the aforementioned macro-modelling approach and the fracture strain for the linear stitches was also selected as 0.33 which were close to the measured test data. Figure 12c shows the load–displacement curves obtained from 5 mm and 10 mm linear seam macro-model simulation and lap shear tests. In case of the 5 mm linear seam, the maximum load obtained from the simulation was 540.1 N which was 37.6 N or 6.9% higher than test data. Similarly, for the 10 mm linear seam, the predicted and actual test loads were 869.35 N and 837.95 N, respectively. Therefore, the predicted value was 3.7% higher than the actual test data. Additionally, Figure 12d represents the sequences of base material failure from the 10 mm linear seam simulation at specific grip displacements. Both the simulations show that the laser macro-modelling approach can be used to predict the joint strength at the early design stage. This can assist design engineers to decide the shape and size of the laser micro-joints as per the strength requirements.

## 6. Conclusions

This paper investigated a macro-modelling approach to represent laser micro-welds for electric vehicle battery application where the macro-model can be used to integrate with a large-scale structural model. Laser micro-joints were produced using copper and Hilumin steel of 0.3 mm tabs in lap shear and T-peel configurations. This study has demonstrated the following:

- Macro-modelling of laser micro-joints predicts similar load–displacement behaviour to that obtained in physical tests.
- Joint fusion zone characteristics are suitable for confirming weld quality and can be further used to obtain knowledge about hardness changes for identifying the key flow stress behaviour of the fusion zone.
- Lap shear and T-peel tests confirmed that macro-model can be used for predicting the maximum load and failure in a computationally efficient way.
- By using the macro-model, maximum joint strength can be predicted within ±7% or lower of the experimentally obtained maximum load. However, further investigations are required to quantify the localised fracture strain based on the weld shape/geometry.

In conclusion, this paper has confirmed the suitability of macro-modelling for predicting joint strength at the early design stage. Further work is needed to integrate the macro-model in a battery module to replicate the accurate behaviour of the battery module.

## Figures and Tables

**Table 1:** Details of materials used in this study.

Material	Specification	Chemical Composition (wt.%)
Steel-Hilumin	DC04 LC; EN 10139/10140	C = 0.047, Mn = 0.235, P = 0.011, S = 0.010 Al = 0.059, Si = 0.002, Bo = 0.0019 Fe-balance
Copper (Cu)	C101S; BS 2870	O = 0.008, Pb = 0.001, Bi = 0.005, Cu-balance

**Table 2:** Physical and mechanical properties of copper and Hilumin used in this study.

Properties	Copper (C101S)	Hilumin
Density [103 kg/m^3^]	8.92	7.75
Young’s modulus, E (GPa)	130	210
Yield strength (0.2%) [MPa]	117	217
Tensile Strength, UTS (MPa)	239	342
Poisson’s ratio	0.34	0.29

**Table 3:** Laser welding parameters and their ranges.

Category	Parameter	Range
Wobble parameter	Frequency	1–1000 Hz
	Amplitude	0.1–0.9 mm
Laser parameter	Power	1–100% (1.5 kW peak power)
	Frequency	1–100 Hz
	Pulse on time	1–10 msec
Machine parameter	Speed	1–1500 mm/min
	Focus range	±1.0 mm

**Table 4:** Laser welding process parameters used in this study.

Parameter	Range
Power	30% (of 1.5 kW peak power)
Welding speed	1500 mm/min
Pulse on time	3 msec
Pulse frequency	30 Hz

**Table 5:** Additional material data and model specific details used to develop the FE model.

Properties	Description/Value
Element type	Discrete—for spring-based load cell Shell—for upper part, lower part, static grip and moving grip Solid—for fusion zone (i.e., weld)
Elastic spring stiffness (N/m)	100,000.0
Rigid body constraints	Constraints in Y and Z displacement (as X corresponds to grip displacement), Constraints in X, Y and Z rotation
Fusion zone scale factor	1.15
Fracture strain	Selected as per the fracture initiation in the experimental load–displacement curve of the specific test type (i.e., lap shear or T-peel)
Contact between BM and weld	TIED_SURFACE_TO_SURFACE (as defined in LS-DYNA)

## Data Availability

Data sharing is not applicable to this article.
